# The Clot Thickens: COVID-19-Related ST-Elevation Myocardial Infarction in the Setting of Recent Boosters

**DOI:** 10.7759/cureus.36814

**Published:** 2023-03-28

**Authors:** Amar R Patel, Kunal Mishra, Andrew J Ortega, Avani R Patel, Fernando D Segovia, Ruben Montanez, Rakhee Makhija, Chanwit Roongsritong, Jorge C Borges

**Affiliations:** 1 Internal Medicine, Texas Tech University Health Sciences Center El Paso, El Paso, USA; 2 Vascular Medicine, University of Virginia, Charlottesville, USA; 3 Internal Medicine, Texas Tech University Health Sciences Center El Paso, Paul L. Foster School of Medicine, El Paso, USA; 4 Internal Medicine, Meharry Medical College, Nashville, USA; 5 Cardiovascular Medicine, Texas Tech University Health Sciences Center El Paso, El Paso, USA

**Keywords:** pci (percutaneous coronary intervention), covid 19 induced thrombosis, covid-19 vaccine complication, st elevated myocardial infarction (stemi), coronavirus disease-2019 (covid-19)

## Abstract

The coronavirus disease of 2019 (COVID-19) has an array of pathological effects that continue to be discovered. Vaccines against COVID-19 have quickly emerged as our main tool. However, the thrombotic risk of both the virus and the vaccine is yet to be established, let alone together. In this case report, we present a case involving a recently diagnosed COVID-19 patient who developed an ST-elevated myocardial infarction (STEMI) after receiving his booster shot. Our aim is to highlight the standard of treatment outcomes in COVID-19-associated clots, familiarize ourselves with the complexity of the clot burden in a COVID-19-associated STEMI, and illustrate the potential role of the cumulative pro-thrombotic effects of a recent COVID-19 booster with a concomitant symptomatic COVID-19 infection.

## Introduction

The coronavirus disease of 2019 (COVID-19) has an array of pathological effects that continue to be discovered. Vaccines against COVID-19 have quickly emerged as our main tool. However, the thrombotic risk of both the virus and the vaccine is yet to be established, let alone together. Even during the early days of the COVID-19 pandemic, physicians recognized that infection with COVID-19 was associated with thrombotic complications, both arterial and venous [1]. Vaccine availability helped reduce the number of cases, but there were still concerns regarding the possibility of thrombus formation secondary to vaccine administration. Cases were soon reported regarding thrombus formation secondary to the vaccine [2-3]. There is a lack of clarity regarding the relationship between COVID-19 infection and whether the vaccine had a protective effect or contributed to a cumulative thrombotic effect regarding the cardiac outcome. This case report addresses some of those unclear correlations and how they have impacted case management for the involved patients. In the following, we present a case involving a recently diagnosed COVID-19 patient who developed an ST-elevated myocardial infarction (STEMI) after receiving his booster shot.

## Case presentation

A 44-year-old male patient with chest pain presented to our hospital as a code heart for a STEMI with an elevated troponin level of 44.3 ng/mL (the troponin I reference range is 0-0.04 ng/mL). The patient presented in acute distress with chest pain and shortness of breath. His symptoms started two days before admission. The chest pain was gradual in onset, intermittent, substernal in location, described as dull pressure in nature, radiated to the left arm, alleviated by lying down, and associated with mild shortness of breath. The patient took one tablet of ibuprofen, which failed to resolve his chest pain. Due to the persistence of chest pain, the patient was brought to the emergency department.

The physical exam was significant for morbid obesity and bilateral lower extremity edema, with the left lower extremity appearing more edematous than the right lower extremity. Vitals showed a heart rate of 92 beats/min, a respiratory rate of 25 breaths/min, a blood pressure of 132/86 mmHg, a temperature of 36.4 degrees centigrade, and saturation at 97% oxygen saturation on a 2 L nasal cannula. During admission, the patient denied nausea, vomiting, abdominal pain, cough, fevers, chills, or diaphoresis.

The patient had a significant past medical history for hypertension, morbid obesity, obstructive sleep apnea (OSA) on continuous positive airway pressure (CPAP), and a recent COVID-19 diagnosis (diagnosed 12 days prior to presentation). The patient received 3/3 Moderna vaccinations, with the most recent vaccination (booster) one week prior to COVID-19 diagnosis. Based on the presentation, the differential diagnosis included COVID-induced STEMI versus COVID/booster-associated STEMI.

**Figure 1:**
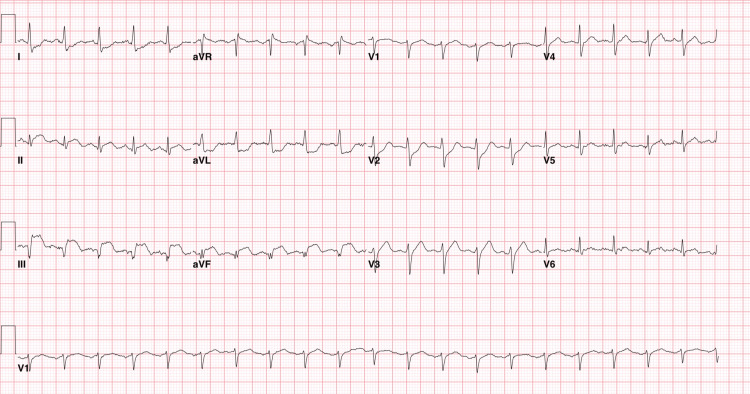
Admission electrocardiogram The admission electrocardiogram demonstrated ST elevation in inferior leads II, III, and aVF suggestive of acute inferior infarct secondary to right coronary involvement. The EKG also demonstrated sinus tachycardia.

Laboratory investigations demonstrated leukocytosis, thrombocytosis, and elevated transaminases. The initial COVID-19 rapid test was negative. An ultrasound of the lower extremities showed an acute non-occlusive thrombus in the midsegment of the femoral vein to the popliteal vein. The electrocardiogram (EKG) revealed ST elevation in inferior leads II, III, and aVF (Figure 1). A transthoracic echocardiogram (Echo) was performed without a bubble study or contrast. The primary care team suspected that the echo would be limited in its findings due to poor visualization of heart structure secondary to the patient’s body weight, which was 166 kg on admission. The Echo demonstrated moderately reduced left ventricular systolic function, an ejection fraction estimated at 30-35%, global hypokinesis with some regional wall motion abnormality suspected, the right ventricle was not well visualized, grossly normal in size with reduced systolic function, valve function not fully evaluated, a dilated ascending aorta, and an echogenic density seen in the posterior part of the pericardial sac, likely fat, but it was unable to rule out clot or effusion (Video 1). Coronary angiography was significant for 100% occlusion of the proximal right coronary artery (RCA) and thrombotic occlusion of the distal left anterior descending artery (LAD) (Videos 2-3). The repeat COVID Biofire test was positive.

**Video 1:** Transthoracic echocardiogram A transthoracic echocardiogram was performed without bubble study or contrast on admission. The primary team suspected that the echocardiogram would be limited in findings due to poor visualization of heart structure secondary to the patient’s body weight which was 166 kg on admission. The echocardiogram demonstrated moderately reduced left ventricular systolic function, an ejection fraction estimated at 30-35%, global hypokinesis with some regional wall motion abnormality suspected, right ventricle was not well visualized, grossly normal in size with reduced systolic function, valve function not fully evaluated, dilated ascending aorta, echogenic density seen in posterior part of pericardial sac, likely fat, was unable to rule out clot or effusion.

**Video 2:** Coronary angiography Coronary angiography was significant for 100% occlusion of the proximal right coronary artery and thrombotic occlusion of the distal left anterior descending artery.

**Video 3:** Coronary angiography Coronary angiography was significant for 100% occlusion of the proximal right coronary artery and thrombotic occlusion of the distal left anterior descending artery.

**Figure 2:**
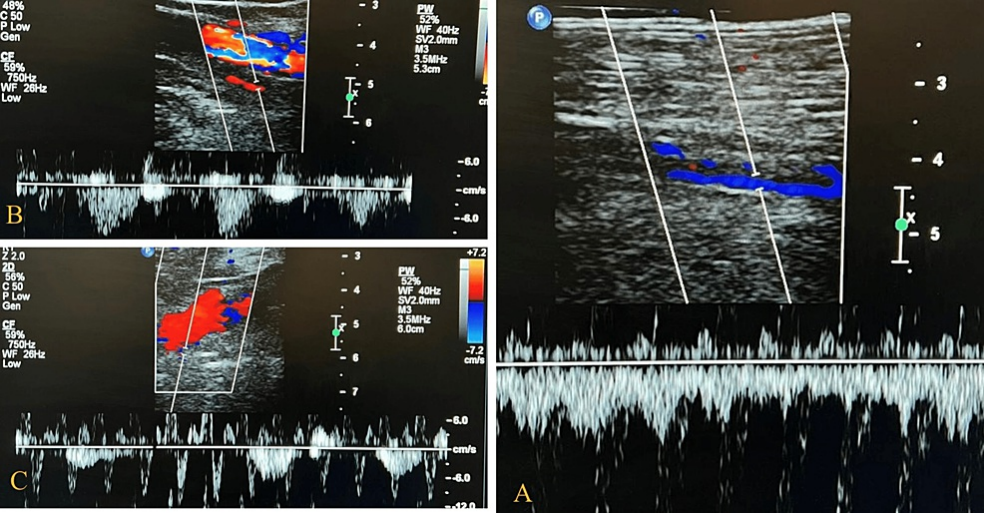
Ultrasound lower extremity venous doppler bilateral (A) Left mid femoral vein demonstrating partial flow, (B) left femoral vein distal, and (C) left popliteal vein. After the percutaneous coronary intervention procedure, the patient had complaints of left leg pain. An ultrasound of bilateral lower extremities revealed an acute left lower extremity deep venous thrombosis extending from mid femoral to the popliteal vein. Note the mid femoral vein demonstrated partial flow only.

The patient underwent percutaneous coronary intervention (PCI) with left heart catheterization and was noted to have severe thrombosis of the RCA and distal LAD. After multiple attempts to restore flow, a twin-pass catheter was passed across the proximal RCA lesion, and intracoronary alteplase 4 mg was introduced. This resulted in an improvement from thrombolysis in myocardial infarction (TIMI)-0 to marginal TIMI-1 flow up to the mid-RCA and minimal flow to the distal RCA. Aspiration thrombectomy (Angiojet) was performed, and significant clot burden was maintained in the RCA and distal LAD despite interventions, with the PCI achieving slight success (TIMI-0 flow improving to TIMI-1 flow) (Videos 4-5). Intracoronary alteplase was not used in the LAD because, at that point in the procedure, the patient had exceeded radiological exposure of greater than 5 Gy and was nearing the contrast limit of 290 ml. Upon reaching maximum limits and with no further complaint of chest pain from the patient, the procedure was aborted despite the continued thrombus burden. The primary team decided to schedule the patient for another PCI procedure at a later date. The patient was then placed on an intravenous eptifibatide drip for 18 hours, per oral aspirin 81 mg once daily, per oral ticagrelor 90 mg twice daily, a heparin drip, a transvenous pacemaker (placed in the right lower extremity), and shifted to the cardiovascular intensive care unit (CVICU). Eptifibatide was discontinued the next day, and the transvenous pacemaker was removed. Beta-blockers were initiated once the patient’s heart rate was sustained. After the PCI procedure, the patient complained of left leg pain. An ultrasound of bilateral lower extremities revealed an acute left lower extremity deep venous thrombosis (DVT) extending from the mid-femoral to the popliteal vein (Figure 2).

**Video 4:** Coronary angiography Coronary angiography was performed and demonstrated severe thrombosis of right coronary artery and distal left anterior descending artery.

**Video 5:** Coronary angiography Coronary angiography was performed and demonstrated severe thrombosis of right coronary artery and distal left anterior descending artery.

Three days later, a repeat right and left coronary angiogram was performed with right radial artery access. Findings included showed residual organized thrombus in the mid-distal RCA and right posterior left ventricular branch (RPLV), with flow further improving to TIMI-2 flow. Additional findings include minimal residual thrombus found in the distal LAD, with flow improved to TIMI-2 flow (Videos 6-7).

**Video 6:** Coronary angiography Three days after the initial percutaneous coronary intervention, a repeat right and left coronary angiogram showed improved flow in right coronary artery and distal left anterior descending artery.

**Video 7:** Coronary angiography Three days after the initial percutaneous coronary intervention, a repeat right and left coronary angiogram showed improved flow in right coronary artery and distal left anterior descending artery.

Given the patient’s elevated inflammatory markers (C-reactive protein greater than 18 mg/dl, ferritin 585 ng/ml), COVID was suspected to be the cause of the clot burden. The patient reported previous exposure to a COVID-positive contact and received the messenger RNA vaccine booster around the same time. After stabilization and discharge, the decision was made to heparin bridge to warfarin with dual antiplatelet therapy. An angiotensin-converting enzyme (ACE) inhibitor was added for further optimization of the heart failure medication. The patient was then followed up by cardiology outpatients and continued on warfarin, aspirin, and ticagrelor.

After discharge, there were multiple follow-up appointments with cardiology. Two weeks after discharge, the patient was seen, and the plan of care was to continue aspirin 81 mg once daily for one month, ticagrelor 90 mg twice daily, and warfarin for a goal international normalised ratio (INR) of 2-3. Three months later, the treatment plan changed. The new plan was to continue the aspirin 81 mg once daily for a minimum of one year, add clopidogrel 75 mg once daily for a minimum of one year, continue the warfarin therapy for a total duration of six months, and stop the ticagrelor due to the patient developing nosebleeds. Six months after PCI, the patient underwent another ultrasound doppler of bilateral lower extremities and found no clot. Warfarin was discontinued. A repeat transthoracic echo was ordered and found that the left ventricular systolic function was normal, the ejection fraction improved from prior levels of 30-35% to 60-65%, the right ventricular systolic function was normal, and no clot or effusion was noted. The decision was made to continue dual antiplatelet therapy for at least one year minimum and then discontinue only aspirin. Clopidogrel was continued for a few additional three months, and after the patient was reassessed by the cardiology team, the clopidogrel was discontinued. The patient continued additional follow-up with the primary care team.

## Discussion

Since the start of the pandemic, the incidence of cardiovascular diseases associated with COVID-19 has risen. COVID-related STEMI, among other cardiac complications, continues to be found nationwide, with thrombotic complications becoming increasingly apparent as a likely etiology [4]. Even with clinical evidence increasing, a clear cause has not yet been identified. Current consensus stipulates that the reason for COVID-19’s coagulopathy may be multifactorial, with endothelial dysfunction, neutrophil extracellular traps, platelet activation, ACE-2 receptor infection, and a severe inflammatory response possibly all playing a role [5-7]. As seen in our case with the presentation of both STEMI and DVT, the coagulopathy secondary to COVID-19 extends to the arterial and venous circulations.

Furthermore, the literature illustrates that the clot burden associated with COVID-19 can be extensive. A recent single-center study shows significantly higher rates of multi-vessel thromboses, like in our patient [5]. However, our case also highlights two important questions: Does the culprit lesion found in COVID-19 STEMI warrant any special considerations when undergoing PCI? Second, does the association between a recent COVID-19 vaccination and a concurrent COVID-19 infection relate to the type of clot burden in our patient?

The answer to our first question was investigated in a recent single-center study published in 2020 [5]. The inclusion criteria included a spectrum of presentations among COVID-19 STEMI patients. They concluded that the primary PCI in COVID-19-related STEMI patients was more complicated due to the unique burden of the coronary thrombus found. Like the large RCA thrombus in our patient, this study showed that a large thrombus burden with a modified thrombus grade of 4/5 was twice as likely to be found in COVID-19 STEMI, along with a higher rate of multivessel thrombosis, also noted in our case with RCA and LAD lesions. However, this study highlights an important fact reported by our interventionalist: due to the angiographic complexity of the clot burden, operators had enhanced use of glycoprotein IIb/IIIa inhibitors, intracoronary alteplase, and thrombectomy. Additionally, despite multiple attempts at recanalization, there was a marginal improvement in coronary artery perfusion from TIMI 0 to TIMI 1, which was interesting enough to resolve our patient’s chest pain. Our case also further demonstrated the impact of placing the patient on aspirin and ticagrelor and later adding clopidogrel and stopping ticagrelor. Our case showcased the difficulty that may arise when performing a PCI on COVID-19-associated STEMI patients and the possibility of limited correlation with coronary artery flow with the patient's presentation. Despite multiple attempts at recanalization, a large clot burden remains, reinforcing the notion that operators should be aware of the potential challenges when performing PCI on COVID-19-related STEMI.

While COVID-19 may induce an intensive inflammatory reaction that manifests as thrombotic events across various vascular beds, the question remains if the concurrence of a recent vaccination prior to/during infection may impact the type of clot burden. While studies have documented the incidence of myocarditis after any dose of the COVID-19 vaccines, the risk of cardiac complications after a COVID-19 infection was found to be worse in a recent head-to-head comparison [8]. However, no study has investigated whether the concurrence of recent COVID-19 vaccination with a simultaneous COVID-19 infection leads to worse outcomes. Based on our literature review, this is the only case documenting the phenomena of a recent COVID vaccination and COVID infection with STEMI presentation. Thus, we cannot make any resolute conclusions regarding the role both the COVID-19 vaccination and the COVID-19 infection played in the cardiac clinical presentation and outcomes of this patient.

## Conclusions

As the prevalence of COVID-19 decreases, more studies will continue to investigate its thrombotic risk. Studies have already shown the correlation between higher thrombus burden and poorer outcomes in patients presenting with STEMI and COVID-19. Whether the role of a very recent COVID-19 vaccination in the presence of a COVID-19 infection may have a cumulative thrombotic effect or protective effect on the cardiac outcome is yet to be seen. The increased clot burden could also be multifactorial given the other patient factors, such as obesity. Due to the increasing number of cases that present this way, it becomes important to finalize newer management strategies for these patients. The above case underwent multiple recanalization attempts and benefited from a multimodal strategy that included intracoronary alteplase, eptifibatide, and post-discharge dual antiplatelet therapy. This case highlights the challenges associated with treating an extensive clot burden in the context of the high inflammatory state of COVID-19. Further research is required to understand how this extensive clot burden develops and if aggressive antithrombotic therapy or varying PCI techniques should be implemented.
